# Integrative Therapies in Wound Healing in Small Animals: An Approach Beyond Traditional Medicine

**DOI:** 10.3390/vetsci13050418

**Published:** 2026-04-24

**Authors:** Jorge Kauã Vila Real Sampaio Santos, Esther Daniela de Sousa Costa, César Carneiro Linhares Fernandes, Annice Aquino Cortez, Arícia Débora Vasconcelos Fonsêca, Rodrigo Fonseca de Medeiros Guedes, Paulo Ricardo Monteiro Araújo

**Affiliations:** 1 Center of Health Science, University of Fortaleza, Fortaleza 60811-905, CE, Brazil; estherdaniela72@gmail.com (E.D.d.S.C.); cesar.fernandes@unifor.br (C.C.L.F.); annice.cortez@unifor.br (A.A.C.); aricia.fonseca@unifor.br (A.D.V.F.); ricardomont.mv@gmail.com (P.R.M.A.); 2 Course of Veterinary Medicine, Education, Sciences and Technology Center of the Inhamnuns Region, State University of Ceará, Tauá 63660-000, CE, Brazil; rodrigo.guedes@uece.br

**Keywords:** wounds, integrative medicine, propolis, medicinal plants, ozone, light therapy

## Abstract

The skin represents the largest organ in the body and functions to protect internal tissues from damage and infection. When wounds in small animals do not receive proper management, they may progress to chronic conditions, resulting in pain, delayed healing, and impaired well-being. Although conventional treatment mainly includes the use of topical antimicrobial agents and anti-inflammatory drugs, integrative veterinary medicine has been considered a promising complementary approach to enhance tissue repair. In this context, this study aimed to review non-conventional therapies applied to wound management in small animals, focusing on ozone therapy, light therapy that stimulates cellular activity, herbal medicine, and apitherapy, especially propolis. Overall, the analyzed studies indicate that ozone may contribute to microbial control and modulation of the immune response; light therapy may stimulate cellular activity and collagen production, promoting healing; medicinal plants present antioxidant and anti-inflammatory effects; and propolis demonstrates antimicrobial and regenerative properties. Thus, when responsibly applied and supported by scientific evidence, these approaches may complement conventional therapy, broaden clinical possibilities, and contribute to improved recovery and quality of life in animals.

## Simple Summary

The skin is the body’s largest organ, acting as a protective barrier against injuries and infections. In small animals, untreated wounds can become chronic, causing pain and delayed healing. Conventional treatments include topical drugs, but integrative therapies can complement them by supporting tissue repair. This review focused on ozone therapy, light therapy, herbal medicine, and propolis. Ozone helps control microbes and supports the immune response; light therapy stimulates cells and collagen production; medicinal plants act as antioxidants and reduce inflammation; and propolis promotes antimicrobial and regenerative effects. When applied responsibly and guided by evidence, these therapies can safely complement conventional treatments, expand clinical options, and improve recovery. Their use also helps reduce reliance on antibiotics, promoting more sustainable care. Overall, these approaches can enhance the health and quality of life of pets, while supporting veterinarians in providing safer and more effective wound management.

## 1. Introduction

The skin is the body’s largest organ and serves as a protective barrier for internal organs, while also performing essential functions such as regulating temperature, detecting sensations, and supporting the immune system [1]. Its structure depends on the coordinated interaction between the different layers, including the epidermis and dermis, as well as associated structures like hair follicles and glands. These components work together to maintain the skin’s integrity and ensure it can effectively protect the organism and support overall physiological processes [2].

In this context, a wound is defined as a lesion characterized by the loss of the anatomical integrity of a biological tissue [3]. According to their morphology and mechanism of trauma, wounds may be classified as open or closed and further subclassified as abrasion, avulsion, incision, laceration, puncture wound, and contusion [4]. Thus, numerous factors may interfere with the repair of injured tissue, such as the extent and vascularization of the affected area and patient comorbidities [5]. When not properly managed or when neglected, an acute lesion may evolve into a chronic condition and struggle to progress adequately through the standard stages of the healing process [6].

Tissue healing occurs in the following phases: hemostatic, inflammatory, debridement, proliferative, and remodeling. Immediately after injury, hemostasis occurs through platelet migration and aggregation. The inflammatory and debridement phases are characterized by increased vascular permeability, allowing the recruitment of immunocompetent cells to the injured tissue, resulting in pain, redness, and edema. Approximately three days after injury, the proliferative phase begins, marked by fibroplasia, angiogenesis, and epithelialization. Finally, the remodeling phase is essential for the reorganization and strengthening of newly formed collagen [7].

It is known that the initial management of wounds consists of covering the lesion with a clean and dry bandage immediately after the injury or upon the animal’s arrival for care, in order to minimize contamination and control possible hemorrhage. Subsequently, clipping of the affected area and debridement of devitalized tissues are performed, an essential step for removing necrotic material and optimizing the healing process. Thereafter, the wound should be irrigated with appropriate solutions to reduce microbial load and remove debris [8].

Conventional drug therapy recommends the use of topical antimicrobial ointments in open wounds to reduce microbial proliferation and prevent infection [4]. In cases of infected wounds, broad-spectrum systemic antibiotic therapy is the treatment of choice. However, the continuous use of antibacterial agents may cause pathogenic bacteria and sometimes the normal microbiota to become resistant, resulting in delayed and difficult healing and consequently increasing treatment costs [9].

In this scenario, integrative medicine emerges as a multimodal approach that complements conventional medicine with non-traditional therapies, assisting in pain control, modulation of inflammation, antimicrobial effects, and tissue regeneration, all of which are essential for lesion repair [10]. Nevertheless, among pet owners and veterinarians, there is still a lack of knowledge, skepticism, and prejudice regarding various integrative therapies, despite the growing body of evidence demonstrating the benefits of integrative medicine in promoting animal health [11].

Therefore, understanding the available therapeutic options is essential for effective wound management. The integration of conventional and alternative methods broadens treatment possibilities and reinforces the importance of integrative medicine in clinical practice. This approach seeks to associate complementary therapies supported by scientific evidence, contributing to the safe and efficient recovery of patients. Moreover, it becomes indispensable in light of the excessive use of antibacterial agents and the increasing risk of microbial resistance, issues that demand sustainable therapeutic alternatives. Against this background, this study seeks to encourage further investigation into integrative medicine, presenting it as a promising alternative to reduce such dependence through safe and effective therapies that enhance tissue healing.

## 2. Materials and Methods

This study consisted of a descriptive scientific literature review based on the analysis and interpretation of previously published scientific studies on the proposed topic. A bibliographic search was conducted in the databases Google Scholar, PubMed, and Web of Science, among others, as well as in relevant veterinary academic textbooks. Only scientific articles and books published between 2020 and 2025 were included. The search addressed current alternative methods for the treatment of wounds in small animals, in addition to topics such as skin histology, phases of wound healing, and wound management. The following keywords were used during the search: “wounds”, “integrative medicine”, “propolis”, “medicinal plants”, “ozone”, and “light therapy”. Publications in peer-reviewed scientific journals and academic books relevant to Veterinary Medicine that addressed the wound healing process and therapeutic approaches used in animal wound healing were included. The exclusion criteria adopted included undergraduate thesis projects, theses, dissertations, academic papers, and publications not pertinent to the topic.

## 3. General Aspects of the Skin and Its Clinical Management

### 3.1. Skin Integrity

**Figure 1:**
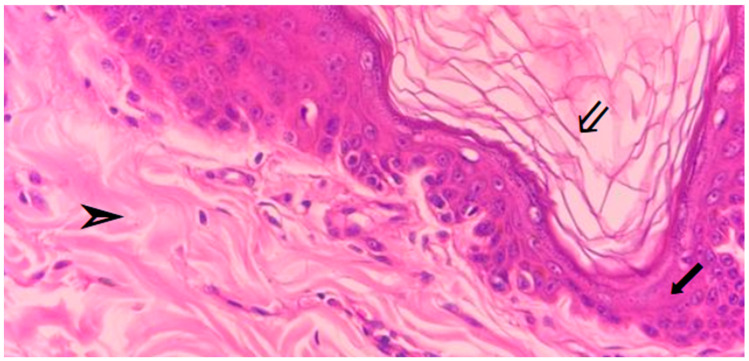
Histological section of canine skin at 40× magnification, showing the epidermal (⇙), dermal (⬋), and hypodermal (➣) layers (original photo by Jorge Santos).

Covering the body, the skin serves as its main barrier, presenting hirsute areas (with hair) and glabrous areas (without hair). It is composed of the epidermis, dermis, subcutaneous tissue (hypodermis), and appendages (hair follicles and glands), as shown in Figure 1. Its thickness varies according to the region and species: it is thicker on the 107 dorsum and lateral limbs, and thinner on the ventral region and medial aspect of the thighs [12].

The epidermis forms the skin’s outer layer and is made of stratified squamous cells, serving as the body’s primary physical barrier. Beneath it lies the dermis, which provides most of the tensile strength and elasticity of the skin and is formed by connective tissue. Additionally, the dermis contains cutaneous appendages, nerves, blood vessels, and lymphatic vessels. The subcutaneous tissue (adipose panniculus) corresponds to the deepest and thickest layer, whose main functions include thermogenesis and energy storage (Figure 1) [13].

A wound is an interruption in the continuity of a body structure, generally the skin, and may be open, with disruption of the surface, or closed, as in hematomas and contusions [14]. Various causes may lead to discontinuities in the skin, such as surgical procedures and high-energy trauma [1].

Wounds may be classified into different types, each with specific characteristics. Abrasion is characterized by a superficial lesion caused by friction or shearing, whereas laceration corresponds to the rupture of the skin and underlying tissue. Avulsion occurs with the separation of tissues at their point of attachment, which may result in flaps or, in limbs, present as a degloving injury. Puncture, or penetrating wound, results from the action of sharp objects or projectiles. Crushing injuries are often associated with other types of lesions and are usually accompanied by deep tissue damage. Finally, a burn consists of a cutaneous injury of variable thickness caused by heat or chemical agents [8].

### 3.2. Physiology of Tissue Repair

Tissue healing is commonly described as a linear process divided into consecutive phases; however, it consists of reactions that frequently overlap, and more than one phase may occur simultaneously [5]. Didactically, these phases are divided into hemostatic, inflammatory, debridement, proliferative, and remodeling stages [7].

The inflammatory phase begins with hemostasis and occurs immediately after trauma. At this moment, vasoconstriction and platelet aggregation take place as the initial response, lasting approximately 5 to 10 min, followed by vasodilation mediated by vasoactive compounds such as histamine, serotonin, and catecholamines [5]. Subsequently, leukocyte migration to the site of injury begins in order to control infection [15].

Degranulation of platelet alpha granules stimulates the initial migration of neutrophils and, subsequently, macrophages. During this process, several mediators are released, including platelet-derived growth factor (PDGF), transforming growth factor beta (TGF-β), insulin-like growth factor I (IGF-I), epidermal growth factor (EGF), as well as fibronectin, thrombospondin, and factor VIII, all of which are crucial for chemotaxis and the recruitment of defense cells [1].

Debridement is characterized by the removal of necrotic debris, pathogens, and damaged cells from the wound. This process occurs through the action of neutrophils, which perform bacterial phagocytosis, and mainly monocytes, which release growth factors and promote the recruitment of mesenchymal cells [15].

After 24 to 48 h in the tissue, monocytes differentiate into macrophages, which secrete collagenases, remove necrotic tissue, and release growth factors responsible for initiating granulation tissue formation and stimulating angiogenesis. Subsequently, lymphocytes act by modulating the pace and quality of tissue repair through the release of soluble factors that coordinate protein production by other cells [8].

The proliferative stage involves the onset of granulation tissue formation, a connective tissue with a granular appearance resulting from processes such as angiogenesis, fibroplasia, and epithelialization, and lasting approximately 2 to 3 weeks. During this period, fibroblasts secrete type I and type III collagen, while epithelialization occurs through the division and migration of epidermal cells [16].

Finally, in the repair or maturation phase, type III collagen is degraded by proteolytic enzymes from macrophages and replaced by type I collagen, which predominates in the skin [17]. Additionally, epithelialization occurs from the wound edges through the migration of keratinocytes toward the center. In larger defects, this process may be delayed and depends on adequate granulation tissue formation [8,13].

### 3.3. Wound Management

Wound management begins with first aid, which consists of applying pressure to reduce bleeding and covering the lesion with a bandage in order to prevent additional contamination and further trauma [18]. Subsequently, wide clipping of the area surrounding the lesion and irrigation with sterile saline solution are recommended to remove debris and reduce the microbial load [19].

Debridement constitutes a fundamental step in wound management and is characterized by the removal of necrotic or devitalized tissues, as well as any possible foreign bodies, in order to promote an environment suitable for healing [8].

After this stage, washing is performed with antiseptic solutions, among which 0.05% chlorhexidine and 0.1% povidone-iodine stand out. Chlorhexidine shows good antibacterial activity, but with limited efficacy against fungi and viruses. Povidone-iodine, on the other hand, has a broader action, being effective against bacteria, fungi, viruses, and protozoa [1]. These antiseptics are useful in the initial control of microbial load; however, they present toxic effects, including cytotoxicity to keratinocytes and fibroblasts, in a manner dependent on concentration and exposure time, and their use is therefore restricted to the initial phase [20,21].

Following antisepsis, the veterinarian must evaluate the lesion to determine whether it should be sutured or allowed to heal by second intention [4]. The professional should take into account the size of the wound, how contaminated it is, and the mechanism of injury [19].

Finally, dressings are applied in direct contact with the wound in order to assist the healing process. Bandages are structured in three layers: the first consists of the primary dressing itself, usually gauze; the second is composed of padding material intended for fluid drainage or the application of pressure; and the third layer is placed externally to secure the dressing and protect the lesion from the external environment [17]. Currently, the progressive replacement of gauze with modern hydrophilic dressings, such as alginates, hydrogels, and hydrofibers, is recommended, as they have a greater capacity to absorb exudate, maintain a moist environment, and reduce trauma to the wound bed. These materials promote drainage by forming gels and conducting exudate to secondary layers, preventing the accumulation of secretions, especially in exudative or cavitary wounds. Adequate drainage directly contributes to the reduction in bacterial load and the efficient progression of healing [22,23,24].

Proper therapeutic management of wounds is of utmost importance for successful tissue repair, and the treatment protocol should be selected based on the type of lesion, its extent, and the degree of contamination, in addition to considering possible concurrent comorbidities of the patient [25].

In cases of contaminated open wounds, the use of topical antibacterial agents is recommended, and in highly contaminated wounds, systemic treatment may be added [1]. Furthermore, for pain control, non-steroidal anti-inflammatory drugs (NSAIDs) are primarily used and may be combined with weak opioids, such as tramadol, in cases of more intense pain [26].

Although topical antibacterials are widely used in wound treatment, their use presents some disadvantages, such as cytotoxicity, hypersensitivity reactions, and the promotion of bacterial resistance. These factors may contribute to wound chronicity, generate stress for the animal, and increase costs for the owner [13].

### 3.4. Bacterial Resistance

Antibacterials exert their action by inhibiting bacterial growth or promoting bacterial death. Each class has a specific mechanism and is classified according to its chemical structure and mode of action [27]. Thus, the indiscriminate use of antibacterial, through inappropriate administration, lack of professional guidance, or failure to complete the prescribed treatment, constitutes a determining factor in the increase in bacterial resistance. This process compromises therapeutic efficacy in subsequent treatments, hinders the clinical management of infections, and contributes to the greater dissemination of infectious agents [28].

Resistance occurs when bacteria acquire genes capable of interfering with the mechanism of action of the antibacterial agent, either through spontaneous DNA mutations or through transformation and plasmid transmission [29]. The mechanisms may be related to the cell wall, such as decreased outer membrane permeability and overexpression of efflux pumps; related to external structures, such as the presence of a capsule and biofilm formation; alteration of the drug target site; or bacterial enzymatic activity against the antibacterial agent. These mechanisms may coexist within the same strain, conferring resistance to different drugs. During replication, genes associated with this resistance may be transmitted to daughter cells and, in some cases, intrinsic mechanisms of one species may be acquired by other bacteria [30].

**Figure 2:**
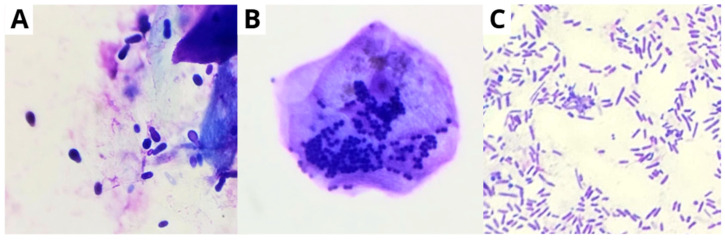
Cytological images of canine skin at 100× magnification: (**A**) showing yeast cells suggestive of *Malassezia* spp., (**B**) cocci observed within a keratinocyte, consistent with bacteria such as *Staphylococcus* spp., and (**C**) demonstrating rod-shaped bacteria (bacilli) compatible with species such as *Escherichia coli* and *Pseudomonas aeruginosa* (Original photos, Paulo Araújo).

Fungi play an active role in infected wounds and are widely recognized as opportunistic pathogens. The presence of an open wound, combined with the use of antibiotics as initial therapy and fungal colonization of the adjacent skin, creates a favorable environment for the development of fungal infections [31]. Species of the genera *Candida*, *Curvularia*, *Malassezia*, *Aureobasidium*, *Cladosporium*, *Ulocladium*, *Engodontium*, and *Trichophyton* are prevalent within the microbial load of chronic wounds in humans. In animals, the yeast *Malassezia pachydermatis* is more commonly reported (Figure 2A), with biofilm production on the skin and in the ears [32,33].

In this context, biofilm stands out as a significant threat to wound healing. Composed of clusters of microorganisms from various species, such as bacteria and fungi, mainly present in the wound bed but also occurring in deeper tissues and along the wound margins. The clinical signs associated with biofilm presence include exudate, necrosis, and desquamation [34]. The bacteria most frequently observed in biofilms include *Pseudomonas aeruginosa*, *Proteus mirabilis*, *Staphylococcus aureus*, *Staphylococcus epidermidis*, *Enterococcus faecalis*, *Streptococcus viridans*, *Klebsiella pneumoniae*, and *Escherichia coli* (Figure 2B,C) [35].

## 4. Integrative Therapies in Wound Healing

### 4.1. Photobiomodulation

Photobiomodulation therapy (PBMT), also known as light therapy, refers to methods that use different types of consistent and controlled light emission with the aim of accelerating the healing process [36]. Among these methods, the use of laser, light-emitting diodes (LEDs), ultraviolet light, and infrared light stands out, being applied in veterinary medicine mainly for managing musculoskeletal and neurological disorders, supporting wound healing, and providing pain relief [37].

An important factor that differentiates the types of light is wavelength, as it determines which tissues will be reached. Shorter wavelengths have lower penetration capacity, whereas longer wavelengths are capable of reaching deeper tissue layers [38].

The mechanism of action of photobiomodulation has not yet been fully elucidated. The main theory suggests that photons, which are particles of light, are absorbed by mitochondrial chromophores in tissue cells [37]. The first chromophore to absorb red light is the enzyme cytochrome c oxidase, which stimulates the production of adenosine triphosphate (ATP), nitric oxide, calcium, and reactive oxygen species (ROS) [39].

At high concentrations, ROS exert cytotoxic effects; however, at low and controlled levels, they perform essential functions, such as promoting cell differentiation, proliferation, and growth, in addition to regulating inflammatory responses [40]. The synthesis of ATP generates additional energy that can be used by the cell to perform various functions, including tissue repair [39]. Moreover, it has been hypothesized that ROS may induce oxidative disruption in certain bacteria, contributing to an antibacterial effect [41].

As a consequence of light stimulation, several growth factors are released, including fibroblast growth factor (FGF) and vascular endothelial growth factor (VEGF). In addition, intracellular signaling pathways are activated, stimulating protein synthesis and epithelial cell proliferation [42]. Thus, photobiomodulation contributes to increased blood circulation, stimulates angiogenesis, promotes collagen synthesis by fibroblasts, and favors osteoblast differentiation, all of which are fundamental elements in the tissue healing process [43].

There is no established consensus on a single protocol for the use of therapeutic light, as variables such as wavelength, affected region, and duration of treatment differ among cases. Therefore, individualization of the protocol for each patient is necessary [37].

#### 4.1.1. Laser

Laser light, due to its monochromatic, coherent, and collimated properties, allows therapeutic energy to be precisely directed to specific areas of the body [44]. Therapeutic lasers emit light at wavelengths ranging from 600 nm to 1200 nm and offer different dosing possibilities (J/cm^2^) [45].

The ideal dosage varies according to the particularities of each patient, since the affected tissue must receive an adequate amount of light energy for therapeutic effects to be achieved. In general, more superficial tissues respond well to doses between 1 and 4 J/cm^2^, whereas deeper tissues require values between 8 and 20 J/cm^2^. In the treatment of open wounds, the laser should not come into direct contact with the lesion, and a dosage between 2 and 8 J/cm^2^ is recommended [44].

A clinical trial demonstrated that the application of therapeutic laser on chronic wounds, at a dose of 4 J/cm^2^, produced superior results compared to the control group that did not receive photobiomodulation. A significant reduction in lesion size was observed, highlighting the potential of laser therapy as an adjuvant treatment to accelerate the healing process in dogs [46].

In a study involving dogs and cats, conducted to evaluate the effect of laser therapy on the healing of surgical wounds, it was observed that the treated areas showed better clinical progression compared to the control areas, with reduced skin thickness, faster resolution of hematomas, greater tissue elasticity, and less fluid accumulation, suggesting a beneficial effect of laser therapy on the tissue repair process [47].

In geriatric patients, the healing process tends to be slower and more prone to complications. A similar situation occurs in individuals with comorbidities that interfere with tissue repair, such as diabetes mellitus, in which laser therapy presents itself as an adjuvant therapeutic to assist in more complex cases [5].

#### 4.1.2. LED’s

LEDs possess the wavelength and power required to assist in the treatment of soft tissues. Unlike laser light, LEDs do not emit consistent and collimated light, which results in a non-homogeneous distribution of light within tissues and lower penetration depth. However, they offer advantages such as a broader illumination area, ease of use, and lower cost compared to laser therapy [45].

There are different types of LEDs, including red, blue, yellow, green, orange, violet, and infrared [48], with red light LEDs, at wavelengths between 680 and 880 nm, being the most recommended for wound healing [49].

In a study on wounds infected with *P. aeruginosa*, it was observed that the use of blue LED resulted in a significant reduction in lesion area, purulent exudate volume, and bacterial colony formation compared to the control group, demonstrating the effectiveness of phototherapy in the healing of infected wounds [41].

### 4.2. Ozone Therapy

Ozone (O_3_) is a triatomic form of oxygen with a dynamically unstable structure. It is colorless, has a pungent odor, and is explosive in its liquid or solid state [50]. Its therapeutic potential has been recognized since the 19th century, initially in the disinfection and healing of severely infected wounds [51].

Ozone therapy is an adjuvant therapeutic practice that uses a mixture of oxygen and ozone, composed of 95% to 99.95% oxygen and 0.055% to 5% ozone, obtained through a certified medical device, and employed in the treatment of a wide variety of diseases [52]. Among the factors supporting the adoption of this therapeutic modality are the reduction in antibacterial resistance during prolonged treatments, as well as the potential adverse effects and costs associated with conventional medications [53].

Historically, the first description of ozone dates back to 1785, when the Dutch physician Martin van Marum reported the presence of an “electrical odor” during experiments involving electric spark discharges in oxygen. It was only in 1840 that the chemist Christian Friedrich Schönbein discovered that the odor was not due to electricity but to a gaseous product formed during the electrical procedure. Schönbein named this substance “ozein,” a Greek term meaning “smell” [50,54].

Ozone has a high oxidative potential, reacting with organic and inorganic compounds until complete oxidation occurs. It decomposes rapidly, generating oxidative byproducts such as reactive oxygen species (ROS) and lipid oxidation products (LOPs). These compounds play a fundamental role in modulating physiological responses by stimulating the release of cytokines and growth factors, promoting immune responses, and accelerating tissue repair [55,56].

ROS restore, in erythrocytes, the rheological properties of blood and aerobic glycolysis through the production of 2,3-diphosphoglycerate, enhancing oxygen transport and adenosine triphosphate (ATP) production by stimulating the Krebs cycle and increasing oxidative carboxylation of pyruvate. Additionally, ROS activates macrophages, neutrophils, and platelets, stimulating the release of autacoids and growth factors. LOPs, in turn, induce endothelial cells to produce nitric oxide at therapeutic levels, contributing to the correction of disorders in various organs and systems. They also promote the release of stem cells from the bone marrow and regulate antioxidant enzymes and cytokines across multiple tissues [57,58].

The antimicrobial effect of O_3_ occurs through oxidation of cell membranes or walls. Upon contact with the gas, a biochemical reaction is triggered that increases the permeability of the bacterial cytoplasmic membrane, culminating in coagulation of the microorganism cytoplasm [53,54].

In fungi, the mechanism is similar, O_3_ promotes oxidative damage to cellular membranes, compromising structural and functional integrity. This effect has been demonstrated through fungicidal activity and inhibition of sporulation in three clinically relevant dermatophyte genera: *Epidermophyton*, *Microsporum*, and *Trichophyton* [36]

Ozone is a natural molecule produced by activated neutrophils during antibacterial activity. Additionally, topical administration of O_3_ at concentrations of 60–80 μg/mL presents a strong bactericidal effect [59].

A series of seven feline cases with extensive limb or distal region lesions treated with a combination of reconstructive surgery and ozone therapy was reported. In all patients, initial clinical stabilization, thorough wound cleaning, debridement, and monitoring of granulation tissue progression were performed. Antibiotic therapy was administered for 5 to 10 days, according to individual case requirements, and faster graft integration, reduced antibiotic dependence, and absence of significant infectious complications were observed [60].

Ozone exerts anti-inflammatory action by inhibiting activation of the nuclear factor kappa B (NF-κB) pathway, resulting in reduced synthesis of pro-inflammatory cytokines such as IL-1, IL-2, IL-6, and tumor necrosis factor alpha (TNF-α). Simultaneously, it increases the production of anti-inflammatory cytokines, including transforming growth factor beta (TGF-β), IL-4, IL-10, and IL-13 [61].

Ozone therapy modulates essential transcription factors by stimulating nuclear factor erythroid 2–related factor 2 (NRF2), enhancing antioxidant and anti-inflammatory responses while repressing NF-κB, the primary regulator of inflammation and cellular stress [62].

The immune response triggered by ozone occurs through immunomodulation, promoting the release of antioxidants [36]. O_3_ stimulates the antioxidant system by inducing mild oxidative stress, leading the body to increase production of antioxidant enzymes such as superoxide dismutase and catalase [56]. Furthermore, O_3_ stimulates the synthesis of interleukins, leukotrienes, and prostaglandins and acts on the humoral immune system, promoting proliferation of immunocompetent cells, immunoglobulin production, and functional activation of macrophages [63].

Ozone can be administered systemically or locally. Systemic administration is primarily performed through ozonated autohemotherapy, which consists of incorporating a controlled concentration of oxygen and ozone into a blood sample that is subsequently reinfused into the patient [64]. Approaches such as the infusion of physiological saline, which promotes increased oxidative activity in peripheral tissues and reduces free radicals in the cardiac, renal, and hematopoietic systems, favoring perfusion and cellular regeneration, as well as the rectal and intravaginal routes, are mentioned in the veterinary literature as possible forms of ozone therapy administration [54,65]. However, some methods, such as blood transfusion and intravaginal application, lack updated publications in small animals, with most evidence based on extrapolations from human techniques or general reviews, highlighting the need for controlled and standardized studies [66,67].

With regard to the safety and physiological effects of systemic ozone therapy, experimental studies have demonstrated promising results. In a study with 10 healthy dogs, rectal ozone insufflation at a dose of 100 µg/kg, administered weekly for 1 month, induced transient oxidative stress followed by a compensatory antioxidant response, without causing relevant clinical or laboratory alterations, demonstrating a favorable safety profile when applied in a controlled manner (Table 1) [68].

Furthermore, clinical evidence reinforces the therapeutic potential of the technique in infectious conditions. In a clinical report on canine parvovirosis, systemic ozone therapy was performed through the intravenous infusion of Ringer’s lactate solution previously ozonated at 41 µg/mL, demonstrating faster improvement of clinical signs and a reduction in hospitalization time when compared to supportive treatment alone (Table 1) [69].

Local administration, mainly used for skin lesions, dermatitis, and infections, can be performed with ozonated water or oil applied directly to the lesion in different pharmaceutical formulations, or through the O_2_–O_3_ gas mixture. This is generally applied in a closed system, such as bagging, in which the limb is placed inside a sealed plastic bag inflated with gas for approximately 10 min, or cupping, which uses a glass cup over the lesion and is indicated for areas where bagging is not feasible, such as facial lesions [36,63,64].

In a case series involving four cats with extensive wounds undergoing surgical reconstruction, ozone therapy was used in the pre- and postoperative periods through local techniques, such as washing with ozonated solution, bagging, and perilesional infiltration, contributing to adequate wound bed preparation and favorable healing progression, without relevant complications, although the results are limited by the absence of a control group (Table 1) [70].

In a clinical report, a dog with cutaneous necrosis resulting from loxoscelism was treated with an integrative ozone therapy protocol associated with laser therapy for 10 consecutive days. The protocol included ozonated oil twice daily, daily bagging for 10 min, and cupping on alternate days, in addition to daily laser therapy. According to the authors, the association of these techniques promoted improvement of the wound bed, with reduction in inflammation and bacterial load. There was a certain delay in healing due to *Loxosceles* venom, with approximately eight months until complete recovery of the lesion. The prolonged healing in this case resulted from the spider bite, and two integrative therapies were applied to accelerate tissue repair. The venom causes tissue destruction by degrading sphingomyelin in cell membranes. The prolonged and recurrent evolution of the lesions, combined with episodes of self-mutilation by the animal, indicates that the management of these injuries requires a more comprehensive approach. The authors emphasize the importance of individualizing protocols, tailored to the particularities of each animal (Table 1) [71].

Despite considerable resistance, controversy, and rejection regarding ozone use among some veterinary professionals, largely due to lack of knowledge about the substance and its therapeutic application, interest has increased, as the search for alternative therapies aims to promote animal welfare while considering cost-effectiveness [72].

This therapeutic modality is used in the treatment of wounds, otitis, inflammation, burns, infectious dermatopathies, acute, chronic, or antimicrobial-resistant infections, ischemic conditions, pain control, as well as in dental procedures and material disinfection [61].

In this context, ozone use optimizes metabolism and oxygenation, exerts bactericidal, fungicidal, and viricidal effects, enhances blood circulation, reduces platelet adhesion, and generates oxidative action on membranes and cytoplasmic components, culminating in neutralization of involved pathogens [73].

The most significant adverse effect associated with O_3_ relates to iatrogenic complications due to inadequate technical training [57]. When therapy is applied excessively and repeatedly, increased desquamation of glandular epithelium may occur. The shed cells tend to cluster into aggregates, which can impair and delay the healing process [65].

The therapeutic effects of O_3_ are dose-dependent, making it essential to adjust ozone product concentrations to prevent toxicity, which occurs when these substances overwhelm the antioxidant system and cause tissue damage [64]. For example, inhalation should be avoided, as pulmonary lining fluid has lower flow and lower antioxidant concentration compared to blood, limiting gas neutralization and causing irritative effects on the mucociliary epithelium and even pulmonary collapse [57,65].

Regarding intravenous administration, ozone rarely causes embolism as it dissolves rapidly in body fluids, unlike oxygen [63]. However, when gas is injected directly into the bloodstream, there is potential for gas embolism, which may be fatal [61].

Ozone therapy is contraindicated in patients with endocrine disorders such as hyperthyroidism, due to ozone’s potential to stimulate thyroid hormone production. It should also not be applied in patients with diabetes, glucose-6-phosphate dehydrogenase deficiency, severe anemia, or active hemorrhage, as rapid oxidative processes may trigger coagulation disorders [65]. Additionally, ozone therapy is contraindicated in pregnant females and animals with myasthenia gravis, and summarize toxicity mechanisms as: formation of free radicals and reactive intermediates, initiation of lipid peroxidation chain reactions, oxidative loss of functional groups and biomolecule/enzyme activity, alteration of membrane permeability and function, and initiation of secondary processes [64].

In this context, there are species-related limitations, since dogs and cats may exhibit distinct responses to oxidative stress. In felines, studies have shown alterations in antioxidant systems, including a reduction in enzymes such as superoxide dismutase and lower total antioxidant capacity under certain clinical conditions, suggesting increased susceptibility to redox imbalances [74]. Therefore, the selection of concentrations and routes of administration should be made with caution. Recent reviews also highlight that, although ozone therapy is considered relatively safe when correctly applied, the lack of standardization of protocols, including dose, exposure time, and frequency, represents a significant limitation for its clinical application in small animals [66,67,75].

### 4.3. Phytotherapy

Phytotherapy, or herbal medicine, consists of the use of plant extracts and other natural compounds for therapeutic purposes in different health conditions [76]. Although it does not completely eliminate pathogens, this practice can act as an adjuvant in the treatment of diseases by strengthening the immune system and assisting in combating microorganisms [77].

In the context of wound healing, herbal products have been used for many years, showing significant effects in treatment. Most of these plants possess antioxidant and anti-inflammatory properties, which contribute to the tissue repair process [78]. Thus, the plants most addressed below for dermatological purposes are: *Aloe vera*, *Calendula officinalis*, *Symphytum officinale* and *Centella asiatica*.

#### 4.3.1. *Aloe vera*

Popularly known as aloe and scientifically referred to as *Aloe vera*, this species is widely recognized for its therapeutic potential and is commonly used in home treatments for skin irritation and hydration, among other applications [1].

The extract of *A. vera* consists of two main fractions: latex and gel [79]. The latex, or sap, corresponds to a bitter liquid that represents 20–30% of the leaf weight and is mainly used for gastrointestinal disorders. Among the components of the extract, chrysophanic acid is known for its fungicidal effects on the skin. In contrast, the gel represents 70–80% of the leaf weight and consists of a viscous, aqueous substance. The gel demonstrates antiviral properties, being effective against strains of herpes simplex virus types 1 and 2, while anthraquinone derivatives present in the gel inhibit viral replication and reduce the cytopathic effect of influenza A virus [80,81].

In the context of antimicrobial action, anthraquinones act similarly to tetracycline by inhibiting bacterial protein synthesis through blocking the ribosomal A site, thereby preventing bacterial growth in media containing the extract [82]. *A. vera* shows significant antibacterial activity against *P. aeruginosa*, an opportunistic Gram-negative bacterium classified by the World Health Organization (WHO) as a Priority 1 organism, for which there is an urgent need for new therapeutic alternatives due to its high antimicrobial resistance [1].

Additionally, formulations containing extracts of this plant species increase collagen activity, enhancing tensile strength and optimizing the process of tissue contraction, as well as stimulating fibroblast proliferation. Moreover, *A. vera* exhibits anti-prostaglandin action by inhibiting thromboxane A synthesized in the dermis after burns [1]. The plant contains the enzyme bradykinase, responsible for the degradation of bradykinin, a mediator of pain, allergies, and acute and chronic inflammation, as well as salicylic acid, which also contributes to the modulation of the inflammatory response [82].

According to a study, topical application of 20% and 40% *A. vera* gel was able to modulate the inflammatory response by reducing concentrations of acute-phase proteins and pro-inflammatory interleukins (IL-1, IL-6, IL-10, and TNF-α), in addition to promoting skin healing in dogs with staphylococcal pyoderma, with no reported topical adverse effects even at the higher concentration. Its topical use has been indicated to promote the healing of acute and chronic dermal lesions, showing good results in wounds resulting from first- and second-degree burns and in patients with hyperglycemic conditions, such as diabetes mellitus (Table 2) [83].

In an experimental study, Wistar rats with full-thickness skin wounds received daily topical application of *A. vera* gel (raw and ethanolic extract) for 21 days. The evaluation included wound contraction, inflammatory biomarkers, and skin histology. The treatment promoted complete healing, normalized skin protein levels, and reduced signs of inflammation, with the ethanolic extract showing greater efficacy, likely due to its higher concentration of bioactive compounds and better skin penetration, without reports of significant irritation, erythema, or necrosis. Despite its efficacy, hypersensitivity, allergy, or effects on pregnancy were not systematically evaluated, indicating that extrapolation to dogs and cats should be done with caution (Table 2) [81].

A separate study evaluated the wound-healing effect of *A. vera* extract in 20 male rabbits with full-thickness skin lesions, in comparison to vitamin E and their combination. Full-thickness wounds were created on the dorsal region of each animal, and the rabbits were randomly assigned to four groups: control treated with saline solution, *A. vera*, vitamin E, and the combined *A.* + vitamin E group, with daily topical application for 14 days. The combined group showed a more significant reduction in wound area compared to the other groups. Histological analysis revealed nearly complete re-epithelialization, denser granulation tissue, and greater collagen deposition, as well as higher biochemical levels of hydroxyproline and antioxidant enzyme activity, suggesting synergistic effects between *A. vera* and vitamin E in accelerating the healing process. However, limitations include the use of a healthy animal model, which may not represent pathological conditions of clinical wounds, and the absence of evaluation of potential adverse skin reactions specific to *A. vera* extract alone (Table 2) [84].

#### 4.3.2. *Calendula officinalis*

Popularly known as calendula, is a plant whose chemical compounds exhibit anti-inflammatory, antimicrobial, anthelmintic, antioxidant, hepatoprotective, and wound-healing properties. These effects significantly contribute to the treatment of various conditions, including gastrointestinal, ophthalmic and dermatological disorders, as well as promoting the repair of cutaneous wounds [85].

The most commonly used parts of *C. officinalis* are the leaves, stem, and flowers, with the latter presenting the greatest phytotherapeutic potential [86]. In Brazil, calendula extract is used in dogs and cats in various forms, such as creams, gels, and tinctures, and for different therapeutic purposes [87].

The compounds responsible for the therapeutic effects of *C. officinalis* include coumarins, carotenoids, amino acids, quinones, saponins, triterpenoids, and flavonoids, with the latter two demonstrating the greatest anti-inflammatory potential [88].

Saponins and flavonoids contribute to the anti-inflammatory and healing action by stimulating fibroblast production and exerting antimicrobial effects through the formation of a protective layer over the wound. They also inhibit histamine release, enhancing healing and analgesic effects, in addition to reducing capillary permeability, thereby inhibiting plasma extravasation into tissues. Carotenoids and flavonoids act as antioxidants, inhibiting free radicals and reactive oxygen species, and also exert immunomodulatory effects by stimulating the immune system. Complementing the anti-inflammatory action, triterpenoids have demonstrated the ability to reduce leukocyte migration to the inflamed area [86,89,90,91].

The effect of an alginate hydrogel containing glycosidic extract of *C. officinalis* was evaluated on the healing of incisional skin wounds in 50 female Wistar rats, which were divided into two groups: a control group, treated with plain alginate hydrogel, and an experimental group, treated with hydrogel enriched with the calendula extract. Topical applications were performed on days 2, 7, 14, 21, and 28, over a total period of 28 days, and the evaluation included histological and cytological analyses, focusing on reduction in inflammatory infiltrate, collagen deposition, and macrophage activation. The experimental group exhibited a significant acceleration of wound healing, evidenced by lower inflammatory infiltrate, higher collagen density, and increased macrophage activation, with no occurrence of cytotoxicity, necrosis, or visible signs of skin irritation. Among the limitations highlighted by the authors were the absence of a clear effect on angiogenesis, the lack of detailed phytochemical characterization of the calendula glycosidic extract, and the need for further studies to fully understand the bioactive compounds and optimize its therapeutic use (Table 2) [92].

The effect of a gel emulsion containing *C. officinalis* oil was evaluated on the healing of skin wounds in female white albino rats, which were distributed into a control group and an experimental group treated with the emulsion. Wound healing was monitored over a period of 14 days, and the results showed that the group treated with the gel emulsion achieved complete epithelial regeneration during this period, being significantly more effective in wound healing compared to the control group. The study did not explicitly report methodological limitations, such as histological assessment, molecular analysis, or long-term follow-up, which restricts interpretation of the underlying mechanisms of the observed effect (Table 2) [89].

Regarding toxicity, the topical use of calendula in animals is generally considered safe and does not cause lethal effects. However, skin irritation or allergic reactions may occur in individuals sensitive to plants of the *Asteraceae* family, such as daisies and chrysanthemums. No recent studies have directly evaluated topical toxicity in animals, but preclinical studies indicate that systemic toxicity is low, even at high oral doses, reinforcing that topical application has a favorable safety profile [93,94].

#### 4.3.3. *Symphytum officinale*

The genus *Symphytum*, belonging to the family Boraginaceae, comprises approximately 40 recognized species. These are perennial plants native to the Euro-Siberian region, currently naturalized in various parts of the world, including South Asia, Australia, Africa, and the Americas [95]. Among its species, *Symphytum officinale* stands out as the most studied, being widely referred to in the scientific literature by the common name comfrey [96].

Different parts of *S. officinale* exhibit significant variations in their phytochemical composition, which directly reflects their biological properties. The roots contain high levels of allantoin and phenolic acids, compounds associated with wound-healing and anti-inflammatory activity, although also related to potential toxic effects. In contrast, the leaves and other aerial parts show a higher predominance of phenolic compounds and flavonoids, mainly contributing to antioxidant and antimicrobial properties. The seeds, in turn, are characterized by a high content of fatty acids, particularly γ-linolenic acid [95,97,98].

The anti-inflammatory effects of *S. officinale* are directly associated with the presence of various bioactive compounds that act by modulating different pathways involved in the inflammatory response. Allantoin stimulates cell proliferation, promoting tissue regeneration. Rosmarinic acid, caffeic acid, and chlorogenic acid, which are phenolic compounds, act by inhibiting the formation of pro-inflammatory mediators, lipoxygenase activity, the release of IL-1β, and nitric oxide production, as well as reducing the expression of pro-inflammatory cytokines, thereby contributing to the observed anti-inflammatory effect. In addition, the naphthoquinone shikonin contributes to the suppression of the transcriptional activation of the TNF-α promoter. The flavonoid rutin also exerts anti-inflammatory effects by reducing TNF-α production, suppressing IL-6 release and inhibiting the activation of the NF-κB pathway [96,99,100].

The antimicrobial effect is related to the bioactive compounds present in its roots, such as phenolic acids and allantoin. The mechanism involves the combined action of secondary metabolites that interfere with microbial growth and metabolism, indicating potential for use as an antimicrobial agent; however, controlled studies are required for standardization and evaluation of efficacy [101].

In an experimental study, the therapeutic potential of comfrey was evaluated in Wistar rats with standardized skin wounds, distributed into groups treated with a base cream (control) and formulations containing 10% and 20% of the plant extract. After 14 days, the extract-treated groups showed faster and more complete wound healing. The 10% group completed the process between days 12 and 14, while the 20% group showed an earlier onset and completion by day 12. In contrast, the control group did not achieve complete healing by the end of the experiment. Additionally, antimicrobial activity was demonstrated against methicillin-sensitive *S. aureus*, methicillin-resistant *S. aureus* (MRSA), *E. coli*, and *P. aeruginosa*. However, the study presents limitations, including the use of a small number of animals and the absence of validation in clinical species, which limits the direct extrapolation of the results (Table 2) [102].

In the context of topical use, *S. officinale* presents a relatively more favorable safety profile compared to oral administration; however, its use is not without risks, as the presence of pyrrolizidine alkaloids may result in systemic absorption, especially when applied to extensive or deep lesions or during prolonged use. Therefore, topical preparations should preferably be free of these compounds and used for limited periods. Nevertheless, the lack of standardization regarding application techniques, extract concentrations, frequency of use, and duration of treatment, combined with the scarcity of comprehensive studies in small animals such as dogs and cats, limits the extrapolation of available data and highlights the need for further investigations to ensure its clinical safety and efficacy [95,96,97].

#### 4.3.4. *Centella asiatica*

Belonging to the Apiaceae family, *Centella asiatica* is widely distributed across Southeast Asian countries, where it is commonly known as Gotu kola, Tiger grass, or Bua-bok. This small perennial herbaceous plant is extensively used due to its extracts positively influencing wound healing [103].

Among the main bioactive compounds of *C. asiatica*, the saponins asiaticoside and madecassoside, as well as their sapogenins, asiatic acid and madecassic acid, stand out and are widely associated with the therapeutic effects of the plant. Among these compounds, madecassoside has been identified as one of the primary contributors to the beneficial effects, by stimulating inflammatory cell infiltration, promoting reepithelialization, and enhancing collagen types I and III synthesis [102]. Asiatic acid is notable for its activity during the proliferative and remodeling phases, stimulating cell proliferation, fibroblast activity, and collagen synthesis, while madecassic acid contributes to anti-inflammatory activity through the negative regulation of cyclooxygenase and inducible nitric oxide synthase (iNOS) expression [104,105]. Asiaticoside also contributes to wound healing, particularly during the inflammatory phase, by reducing pro-inflammatory cytokines and modulating growth factors. Additionally, it stimulates angiogenesis, collagen synthesis, and increases tensile strength, further contributing to the inhibition of inflammatory responses in keloids and hypertrophic scars [106,107].

Additionally, *C. asiatica* exhibits significant antimicrobial activity, being effective against various clinically relevant microorganisms. Reviews have demonstrated antibacterial effects against *S. aureus*, MRSA, *E. coli*, *Streptococcus* spp., *P. aeruginosa* and *Chromobacterium violaceum*. Regarding antifungal activity, the plant’s aqueous extract has shown efficacy against fungi such as *Cladosporium cladosporioides*, species of *Aspergillus*, *Penicillium*, and *Fusarium oxysporum*, as well as others including *Candida albicans* and *Microsporum boulardii*. Both the leaves and roots of the plant demonstrate antimicrobial and antifungal activity [105,106].

In a recent experimental study with *C. asiatica*, it was demonstrated that topical application of the plant accelerates the healing of cutaneous wounds in a diabetic mouse model induced by a high-fat diet and streptozotocin. Thirty male mice were divided into three groups (vehicle, *C. asiatica* at 200 µg/cm^2^, and 300 µg/cm^2^), with full-thickness cutaneous wounds of 6 mm created on their dorsums and treated daily for 9 days. It was reported that by day 7, the treated groups showed significantly greater wound closure compared to the control, with the 200 µg/cm^2^ group exhibiting the highest efficacy. Additionally, increased reepithelialization, greater collagen deposition, and enhanced angiogenesis were observed, along with reduced expression of iNOS and pro-inflammatory cytokines. No significant adverse effects were reported in the animals during the observation period; however, the study had limitations, including the short duration of 9 days, the use of a single diabetic wound model, and the absence of long-term evaluations regarding the quality and functionality of complete wound healing (Table 2) [108].

In another study, the effect of topical application of *C. asiatica* extract on the healing of cutaneous wounds was evaluated in 20 male diabetic rats, induced with streptozotocin and a high-fat diet. The animals were divided into four groups (control and *C. asiatica* gel at 2.5%, 5%, and 10%), with daily application of the gel on the wounds for 14 days, and assessments were made on days 4, 7, 11, and 14. All treated groups showed significant improvement compared to the control, with the 5% gel being the most effective, exhibiting faster wound closure from day 7 and nearly complete healing by day 14. This suggests that the intermediate dose maximizes the plant’s regenerative activity, likely by minimizing cytotoxic effects and promoting optimal tissue response. The authors concluded that topical application of *C. asiatica* accelerates wound healing in diabetic rats; however, they highlighted limitations such as the absence of detailed histological and molecular analyses, the evaluation being restricted to wound length, and the short follow-up period, which did not allow for full assessment of the quality of the regenerated tissue (Table 2) [109].

There is limited evidence in the literature regarding the adverse and toxic effects of *C. asiatica*; however, cases of contact dermatitis have been reported, and it is generally considered safe [104]. Its effects on collagen production, inflammation regulation, and antioxidant activity promote faster and more efficient wound healing. Nevertheless, further studies are needed to better characterize the properties of the extracts used [106].

**Table 2:** Herbal-Based Case Studies.

Herbals	Specie Count	Wound Types	Period	Group-Wise Formula	Results	Authors	Reference
*Aloe vera*	20 dogs, 4 equal groups	Chest, bacterial lesions	2 weeks	Control positive without treatment/*A. vera* 20% gel/*A. vera* 40% gel/Gentamicin 1% ointment	*A. vera* gel 20% and 40% showed therapeutic, antibacterial, and anti-inflammatory effects comparable or superior to 1% gentamicin in dogs with pyoderma	Kamr et al., 2020	[83]
*Aloe vera*	120 rats, 5 equal groups	Dorsal, full-thickness circular excisional	21 days	Normal control (no excision)/Positive control without treatment/Reference control standard ointment/Gel 100% pur/Ethanolic Extract 70%	The ethanolic extract of *A. vera* was the most effective, promoting complete wound healing, followed by the reference control, which also achieved full recovery.	Arafa et al., 2025	[81]
*Aloe vera*	20 rabbits, 4 equal groups	Dorsal, full-thickness circular	14 days	Saline topical/*A. vera* extract 10%/Vitamin E 5%/*A. vera* 10% + Vitamin E 5%	The combined group reduced wound area, more than Aloe vera, vitamin E and control	Khan et al., 2020	[84]
*Calendula officinalis*	50 rats, 2 equal groups	Dorsal, full-thickness circular	28 days	Control with Alginate hydrogel/Calendula Alginate hydrogel	Calendula-alginate hydrogel accelerates wound healing and shows anti-inflammatory effects by day 14.	Posa et al., 2024	[92]
*Calendula officinalis*	Rats, randomly allocated, 2 groups	Perineal, cutaneous	14 days	Control group without treatment/Experimental group with Calendula Oil	The experimental group showed a higher wound healing percentage (29.5%).	Wijayanti & Luthfiyati, 2021	[89]
*Symphytum officinale*	Rats, randomly allocated, 2 groups	Dorsal, excisional full-thickness circular punch defects	14 days	Control group treated with standard and oil-in-water /Oil-in-water cream containing 20% *S. officinale* root extract	Faster and complete healing with 20% comfrey cream, with advanced regeneration observed at 14 days and antimicrobial effect.	Mârza et al., 2024	[102]
*Centella asiatica*	30 mice, 3 equal groups	Dorsal, full-thickness circular	9 days	Control group without treatment/Topical preparation of *C. asiatica* at 200 µg/cm^2^/ Topical preparation of *C. asiatica* at 300 µg/cm^2^	The 200 µg/cm^2^ dose showed the best results, with faster wound closure, greater reepithelialization, collagen deposition, and angiogenesis, outperforming both the control and 300 µg/cm^2^ groups, likely due to reduced cytotoxicity.	Xiao et al., 2025.	[108]
*Centella asiatica*	20 rats, 4 equal groups	Dorsal, full-thickness circular	14 days	Control group without treatment/*C. asiatica* 2.5% gel/*C. asiatica* 5% gel/*C. asiatica* 10% gel	The 5% *C. asiatica* gel showed the fastest wound closure and best collagen and fibroblast response.	Dewi et al., 2023	[109]

### 4.4. Apitherapy

Apitherapy refers to the use of products derived from the honeybee (*Apis mellifera*) for the purpose of preventing or treating diseases and health disorders in humans and animals [110]. It is considered a branch of complementary and alternative medicine and may be integrated with conventional therapies to restore homeostasis and promote health. This approach may employ bee products alone (apimedicine) or in association with medicinal plants (apipharmacopoeia), according to integrative clinical protocols [111].

The products produced by bees include honey, propolis, royal jelly, bee pollen, beeswax, and bee venom, all recognized for containing bioactive constituents with therapeutic potential [112].

#### Propolis

Propolis stands out as a resinous substance collected by bees from plant exudates, leaf buds, and tree sap. In the hive, it is used to seal cracks, smooth internal surfaces, reinforce structural stability, and act as a protective barrier against microorganisms and invaders [113].

Each type of propolis presents a distinct chemical composition influenced by botanical and geographical origin, which impacts its therapeutic efficacy and clinical applications. Among the various types of propolis, the green, red, and brown varieties stand out. Green propolis shows high concentrations of artepillin C and flavonoids, associated with greater antioxidant potential. Red propolis, rich in isoflavones such as vestitol and neovestitol, is notable for its antibacterial activity against *S. aureus* and *E. coli*, while brown propolis, with higher levels of terpenes and flavonoids, also demonstrates antimicrobial action [114].

Its chemical composition is complex and heterogeneous, including flavonoids, terpenoids, phenylpropanoids, and other bioactive secondary metabolites. Evidence indicates that phenolic compounds play a central role in the biological activities attributed to propolis extracts [115].

Several compounds present in propolis contribute to its pharmacological properties. The antimicrobial effect may occur through direct action on microorganisms, altering cell membrane permeability and causing leakage of intracellular components, or through indirect mechanisms such as macrophage stimulation and activation of Th1-type cellular immune responses. Antimicrobial activity is mainly associated with flavonoids such as pinocembrin, galangin, and pinobanksin, as well as caffeic acid esters. Antioxidant activity is related to kaempferol and caffeic acid phenethyl ester, while anti-inflammatory activity involves flavonoids such as quercetin and naringenin and cinnamic acid derivatives, including caffeic acid phenethyl ester, through inhibition of the cyclooxygenase pathway and prostaglandin biosynthesis, neutralization of free radicals, reduction in cytokine production, modulation of nitric oxide synthesis, and immunosuppressive action [110,113].

In addition to antibacterial activity, propolis exhibits antiviral properties against Herpes simplex virus type 1 (HSV-1) and type 2 (HSV-2), as well as antifungal activity demonstrated in an experimental model of vulvovaginal candidiasis, with results similar or superior to nystatin depending on the concentration used [115]. Regarding antiparasitic activity, extracts of green and red propolis demonstrated efficacy against *Leishmania amazonensis*, while green propolis showed additional effects against *Trypanosoma cruzi* [114].

In the context of tissue repair, topical gel formulations containing 1.2%, 2.4%, and 3.6% of dry propolis per total volume of the gel promoted significant improvement in cutaneous wound healing, with the best performance observed at 3.6%, suggesting a possible dose-dependent effect. This healing effect is associated with modulation of extracellular matrix components, including fibronectin, broad-spectrum antibacterial activity, stimulation of neoangiogenesis, and enhanced epithelialization, thereby promoting tissue repair [110].

## 5. Conclusions

Photobiomodulation exhibits considerable therapeutic potential, with notable effects observed both with lasers and LEDs, including the induction of VEGF and FGF, as well as improved granulation tissue formation in chronic wounds. Nevertheless, further updated studies are required, specifically regarding the use of LEDs, whose effects and protocols still lack standardization. Even so, this therapy is consolidated as a modern and sophisticated approach, capable of integrating safety, efficacy, and versatility in the management of wounds and complex lesions.

The application of ozone therapy demonstrates promising adjuvant effects in veterinary medicine. The literature suggests that O_3_ administration can accelerate wound healing, reduce antibiotic dependence, and stimulate tissue regeneration. Ozone therapy was the most frequently reported among the therapies analyzed, with various protocols and techniques described, including systemic and topical administration, highlighting its clinical versatility. Standardization of protocols and the conduction of controlled clinical studies remain necessary to consolidate its efficacy and safety in small animals.

Analysis of phytotherapeutics and propolis reveals beneficial effects on wound healing. Experimental literature demonstrates promising results, such as the antimicrobial effects of *S. officinale* and *C. asiatica*, with reduction in MRSA infection. Clinical translation, however, still presents significant limitations, as many studies are experimental, conducted in healthy or specific animal models, with short observation periods, small sample sizes, and lack of evaluation of certain phytotherapeutics in species such as dogs, cats, or rabbits. For example, *S. officinale* has only one recent study evaluating its wound-healing effect, without relevant clinical data. Safe extrapolation is also hindered by the fact that some of these plants and propolis arestudied in human medicine research, but gaps remain regarding dose standardization, toxicity, and formulations in veterinary medicine.

Therefore, all therapies addressed in this review demonstrate generalized therapeutic potential for wound healing. However, rigorous, standardized clinical studies with histological and functional evaluation of regenerated tissue are required, including investigations in non-model species. Additionally, monitoring through regular cultures and cytologies, as well as the assessment of inflammatory markers, is pertinent to evaluate the antimicrobial and anti-inflammatory effects of these therapies.

## Figures and Tables

**Table 1:** Ozone Therapy-Based Case Studies.

Specie Count	Administration	Period	Group-Wise Formula	Results	Authors	Reference
10 dogs, 3 groups	Systemic/ Rectal ozone insufflation	1 month	Four weekly applications at a dose of 100 µg of O_3_ were performed.	Transient oxidative stress was observed, followed by an antioxidant response.	Oliveira et al., 2024	[68]
4 dogs, 2 equal groups	Systemic/Intravenous infusion of ringer’s lactate	1 week	Control group received supportive treatment (antibiotic therapy, antiemetics, and fluid therapy)/Experimental group received ozonized fluid therapy at a dose of 41 μg/mL and supportive treatment	The experimental group stopped vomiting within 48 h and was discharged between 4.5 and 5.5 days. In the control group, only one animal was discharged after 6.5 days of hospitalization, while the other participant died.	Santos et al., 2023	[69]
4 cats, distinct cases	Topical/ Ozonated solution, *bagging* and perilesional infiltration	3 months	In all four cases, the following procedures were performed: bagging at different ozone concentrations (20–60 μg/mL), perilesional subcutaneous infiltrations (15 μg/mL), and ozonized saline lavages.	The results in all four cases showed progressive and complete wound healing. Initial wounds demonstrated limited epithelialization, but by the end of the follow-up period, all cats exhibited complete epithelial coverage and hair regrowth.	Oros et al., 2025	[70]
1 dog	Topical/Ozonated oil, *bagging*, *cupping* and laser therapy	8 months	Ozone therapy with the application of ozonized oil twice daily, daily bagging for approximately 10 min, and cupping on alternate days, associated with laser therapy performed once a day for about 10 consecutive days.	The multimodal treatment promoted favorable healing with granulation tissue formation. Ozone therapy contributed to improving the wound bed and reducing inflammation and bacterial load.	Pinto et al., 2024	[71]

## Data Availability

The original contributions presented in the study are included in the article. Further inquiries can be directed at the corresponding author.
